# The ‘melanoma-enriched’ microRNA miR-4731-5p acts as a tumour suppressor

**DOI:** 10.18632/oncotarget.10109

**Published:** 2016-06-16

**Authors:** Mitchell S. Stark, Lisa N. Tom, Glen M. Boyle, Vanessa F. Bonazzi, H. Peter Soyer, Adrian C. Herington, Pamela M. Pollock, Nicholas K. Hayward

**Affiliations:** ^1^ Dermatology Research Centre, The University of Queensland, School of Medicine, Translational Research Institute, Brisbane, QLD, Australia; ^2^ QIMR Berghofer Medical Research Institute, Herston, Brisbane, QLD, Australia; ^3^ School of Biomedical Sciences, Institute of Health and Biomedical Innovation, Queensland University of Technology, at The Translational Research Institute, Brisbane, QLD, Australia

**Keywords:** SSX, microRNA, melanoma, PMP22, miR-4731

## Abstract

We previously identified miR-4731-5p (miR-4731) as a melanoma-enriched microRNA following comparison of melanoma with other cell lines from solid malignancies. Additionally, miR-4731 has been found in serum from melanoma patients and expressed less abundantly in metastatic melanoma tissues from stage IV patients relative to stage III patients. As miR-4731 has no known function, we used biotin-labelled miRNA duplex pull-down to identify binding targets of miR-4731 in three melanoma cell lines (HT144, MM96L and MM253). Using the miRanda miRNA binding algorithm, all pulled-down transcripts common to the three cell lines (n=1092) had potential to be targets of miR-4731 and gene-set enrichment analysis of these (via STRING v9.1) highlighted significantly associated genes related to the ‘cell cycle’ pathway and the ‘melanosome’. Following miR-4731 overexpression, a selection (n=81) of pull-down transcripts underwent validation using a custom qRT-PCR array. These data revealed that miR-4731 regulates multiple genes associated with the cell cycle (e.g. *CCNA2*, *ORC5L*, and *PCNA*) and the melanosome (e.g. *RAB7A*, *CTSD*, and *GNA13*). Furthermore, members of the synovial sarcoma X breakpoint family (SSX) (melanoma growth promoters) were also down-regulated (e.g. *SSX2*, *SSX4*, and *SSX4B*) as a result of miR-4731 overexpression. Moreover, this down-regulation of mRNA expression resulted in ablation or reduction of SSX4 protein, which, in keeping with previous studies, resulted in loss of 2D colony formation. We therefore speculate that loss of miR-4731 expression in stage IV patient tumours supports melanoma growth by, in part; reducing its regulatory control of SSX expression levels.

## INTRODUCTION

In our previous study, miR-4731-5p (miR-4731) was identified as a melanoma-enriched (mean 24-fold; *P(cor)*=1.47E-04) miRNA following a comprehensive analysis of a large panel of melanoma cell lines (expressed in 37/55) in comparison with other cell lines derived from solid malignancies (expressed in 5/34) [1]. In a separate study, we also found miR-4731 to be down-regulated (23-fold mean; *P(cor)*=0.004) in metastatic melanoma spread to distant sites (stage IV) compared with loco-regional metastases (stage III) [2]. Moreover, together with the expression of another miRNA (miR-204), miR-4731 was able to distinguish stage III from stage IV tumours with high sensitivity and specificity (AUC=0.89; *p*=0.003, OR=3.0, CI 1.45-6.2) [2].

miR-4731 is located at chr17p12 (chr17:15,154,944-15,155,013) in an intron (>5 kb from intron-exon border) of the peripheral myelin protein 22 gene (*PMP22*), which is commonly associated with hereditary Charcot-Marie-Tooth disease type 1A (CMT1A). *PMP22* is expressed primarily in neural crest derived cell lineages (including melanocytes), however expression of *PMP22* has also been associated with cell proliferation and survival in breast cancer [3–5]. To date no target genes of miR-4731 have been functionally validated.

Given the association of miR-4731 with melanoma in our previous studies, we sought to identify the genes regulated by this miRNA. We employed the optimised biotin-labelled miRNA duplex pull-down procedure [1, 6, 7] to identify binding targets of miR-4731, followed by gene-set enrichment analysis (GSEA) to help elucidate significant pathways and biological processes regulated by miR-4731. A selection of pull-down target genes (n=81) underwent validation via qRT-PCR following over-expression of a miR-4731 mimic in three melanoma cell lines. We report here that miR-4731 has the potential to regulate multiple genes involved in the cell cycle and the melanosome. Importantly, overexpression of miR-4731 inhibits SSX4 protein (pull-down target) resulting in a marked reduction in 2D colony formation in 3/3 melanoma cell lines.

## RESULTS AND DISCUSSION

### The verification of miR-4731 as a melanoma-enriched miRNA

**Figure 1:**
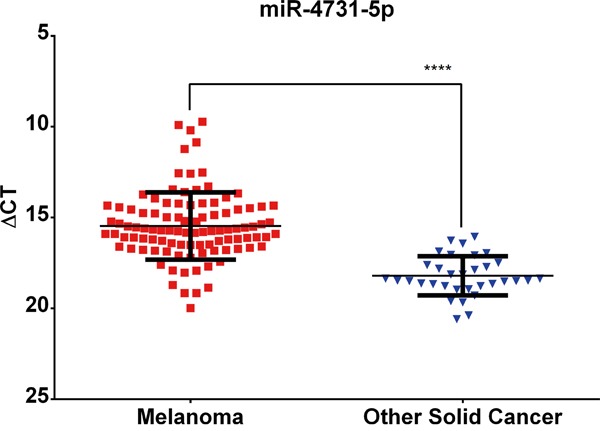
miR-4731-5p expression is significantly (Mann-Whitney U-test; ****= P≤0.0001) associated with melanoma cell lines as compared to other solid cancers ΔCT values are plotted following comparison with endogenous levels of RNU6 assessed in each sample. Error bars represent one SD from the mean.

miR-4731 was identified following a comprehensive miRNA microarray (miRBase v18) analysis of a panel of melanoma cell lines (n=55) compared with other solid malignancies (n=34) [1]. In the current study, the microarray expression data for miR-4731 was validated using qRT-PCR in an extended panel of cell lines derived from melanoma (n=100; including 55 that were initially assayed) and other solid cancers (n=34) (Supplementary Table S1). The mean expression level of miR-4731 is significantly higher (Mann-Whitney U-test; *P*≤0.0001) in melanomas compared to other cancers (Figure 1). The expression of *PMP22* (miR-4731 host gene) was assessed in relation to that of miR-4731 to identify any correlations. In 14/43 (32%) melanoma cell lines with no detectable miR-4731 expression, *PMP22* was expressed above background (data not shown). This is suggestive that miR-4731 is not under the same transcriptional control as its host gene and is independently regulated. In the remaining samples (29/43), there was an inverse correlation observed (Pearson's R^2^= −0.25) which suggests that expression of *PMP22* may be negatively regulated by miR-4731 (data not shown).

### Target gene identification via biotin-labelled miRNA duplex ‘pull-down’ of mRNA transcripts

To identify genes potentially regulated by miR-4731, we used the unbiased biotin-labelled pull-down procedure [1, 6, 7], which harnesses the conventional AGO2-directed binding of the mature miRNA to the mRNA transcript. By modifying the miRNA sequence with a biotin label, miRNA:mRNA bound transcripts can be ‘pulled-down’ using streptavidin-coated magnetic beads. This procedure was applied to three melanoma cell lines (HT144, MM96L and MM253), chosen based on their low, yet detectable endogenous expression of miR-4731, together with transfection ability. As we were looking for enrichment of biotin-labelled transcripts, we only considered transcripts that were up-regulated in each sample compared to the biotin-labelled negative control (Neg-Scr) (see Materials and Methods). There were numerous targets (887-2496 transcripts) specific to each cell line, likely due to inherent differences between them, such as global gene expression profiles and mutational background. Due to these differences we focussed on common transcripts between all three cell lines (Supplementary Figure S1). Obsolete and duplicate transcripts were removed, which left 1154 unique transcripts (Supplementary Table S2) representing 1092 different genes (see Materials and Methods).

### Verification of ‘pulled-down’ genes using prediction algorithms

Full-length (5′ UTR, protein coding sequence, and 3′ UTR) FASTA sequences were collated for each transcript (n=1154) and parsed through the prediction algorithm miRanda-3.3a (see Materials and Methods). All pulled-down transcripts were predicted to be targets of miR-4731 by the program when the binding threshold was set at 100 (data not shown). However by reducing the stringency threshold, the algorithm may allow for an increased number of false positives. The reduced threshold was used to highlight that miR-4731 had the capacity to bind to the transcript given the right biological conditions. It is interesting to note that when the miRanda algorithm was instructed to only call ‘seed’ binding occurrences, believed to be the most common binding type, 505/1154 (44%) transcripts were called. Therefore, the majority of the pull-down (PD) transcripts had a mixture of ‘non-seed’ binding present (e.g. centred, 3′ supplementary, and bulged sites [8–10]), which indicates that there was no bias toward particular sites based on fold enrichment. This observation may in part be explained by experimental bias previously observed with this procedure [6], that the biotin tag on the synthetic miRNA duplexes may actually prevent their 3′ end from binding to AGO [6]. It is believed that the biotin-labelled miRNAs interact more readily with mRNAs and alleviate the reliance on ‘seed’ binding from the 5′ end [6]. We found that 44% of binding sites can be attributed to seed binding, which compares well to a previous estimate of 41% [6]. As previously mentioned, miRanda 3.3a was used to confirm the binding of the PD transcripts using a lower stringency threshold score (100) but if stringent default settings were used, (binding threshold =140) only 668/1154 (58%) of transcripts were called. Other commonly used prediction programs (with default parameters applied) performed poorly when compared to the PD transcripts and miRanda 3.3a; TargetScan (27%), TargetMiner (23%), DIANA-microT-CDS (19%), and miRDB (2%). In the absence of extensive data confirming the specificity of the interactions predicted by miRanda (which was not the focus of this study) we cannot conclude that miRanda is better than the other predictive programs, however it does highlight that multiple algorithms need to be assessed and functional studies need to be performed to validate these findings. On the other hand, the discrepancy observed between the PD transcripts and the prediction algorithms may be due to a high number of false-positives present in the dataset. It has been previously observed that a proportion of PD transcripts may arise via non-specific interaction with the magnetic streptavidin beads, or by association with endogenous biotin, and not the intended biotinylated miRNA [6]. However, as all PD transcripts were compared to those generated using a negative, scrambled sequence, we have reduced the occurrences of false positive PD transcripts, but their presence cannot be completely eliminated.

### Network analysis reveals enrichment for key signalling pathways and gene ontology terms

It is widely accepted that a single miRNA has the potential to regulate a multitude of signalling pathways, achieved by the direct targeting of individual genes, which in turn elicit their function on their next interacting partner. The understanding of a miRNA's target gene(s) is not only important for understanding their contribution to the disease process, it is crucial when it comes to utilizing miRNAs as therapeutic agents [11]. Rather than individual mRNA:miRNA pairings in a signalling pathway, many genes within a pathway/process may undergo regulation by a single miRNA (or cluster).

**Figure 2:**
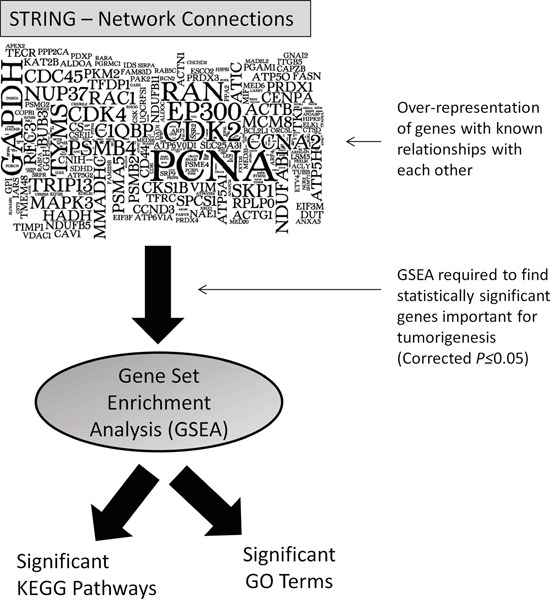
Visualisation of the numerous protein-protein network connections observed after STRING analysis (larger font indicates genes with the most interactions with others in the geneset) and following subsequent enriched analysis, significant (corrected *P*≤0.05) pathways and GO terms were identified.

Of the miR-4731 PD transcripts, we postulated that these genes were not simply randomly selected by the miRNA but may actually form a network of interactions with each other. In steps to identify interacting partners contained within our dataset, we used the network analysis program STRING (v9.1) [12] which identifies known and predicted protein-protein interactions. Using default settings, there were a vast number of interactions (n=4225), with many genes being involved in >1 connection (visualised in Figure 2). To identify the significance of these interactions, gene-set enrichment analysis (GSEA) was next used to identify statistically (corrected Bonferroni P-values; see Materials and Methods) significant pathways (KEGG) and Gene Ontology (GO) terms. The top three KEGG pathways of note were related to ‘Metabolic pathways’ (109 genes; *P(cor)*=8.94E-11) the ‘Cell cycle’ (25 genes; *P(cor)*=9.24E-08) and ‘Oxidative phosphorylation’ (22 genes; *P(cor)*=3.98E-05) (Supplementary Table S3). Notably, of particular relevance to the melanocytic lineage, was the GO term ‘Melanosomes’ (Supplementary Table S3), consisting of 22 miR-4731 target genes (*P(cor)*=3.15E-07).

### Overexpression of a miR-4731 mimic and regulation of pull-down transcripts

Given that 1154 transcripts (1092 genes) were common to all 3 melanoma cell lines, it was not feasible to validate all of these genes via reporter assays, qRT-PCR or Western blotting. We did however assess 81 (7%) transcripts (Supplementary Table S4) using a custom qRT-PCR array (QIAGEN). The criteria for transcript selection are detailed in the Materials and Methods but can be summarised as follows: 1) the host gene of miR-4731, PMP22 was selected to confirm if it was under transcriptional control of miR-4731; 2) all genes that had ≥2 fold enrichment in 3/3 cell lines (n=37); 3) significant pathways were selected based upon *a priori* relevance to melanoma (selected pathways were ‘cell cycle’, ‘oxidation phosphorylation’, and ‘melanosomes’). These pathways had further restrictions imposed in an attempt to be more stringent (n=43) (see Materials and Methods). We over-expressed a miR-4731 mimic or a negative control mimic (Neg-scr), in the same melanoma cell lines (HT144, MM96L and MM253) that were used to perform the pull-down procedure. Supplementary Figure S2 highlights that a consistent over-expression was achieved in all cell lines tested. After a 72 hr transfection, subsequent qRT-PCR revealed that *PMP22* (miR-4731 host gene) was downregulated (1.4-2.2 fold) in 3/3 cell lines upon miR-4731 overexpression (Supplementary Table S4). These data support the previously observed inverse correlation between expression levels of *PMP22* and miR-4731. All other validated target genes related to ‘cell cycle’, ‘oxidative phosphorylation’, and ‘melanosomes’ can be found in Supplementary Table S4 and summarised in Supplementary Table S5: those that were down regulated in 3/3 (n=19), 2/3 (n=12) and 1/3 (n=19); both up and down regulated (n=5); up regulated (n=5); and not observably changed at the mRNA level (≤1.2 fold up or down regulated) (n=21). These data provide validation of the binding effect of miR-4731 to up to 74% of the assessed transcripts. The 26% (21/81) of transcripts that were not observably changed at the mRNA level may be false positives. To confirm the absolute effect on the target genes, assessment at the protein level must occur to confirm these findings.

### Key members of the melanosome and cell cycle pathways are regulated by miR-4731

#### Melanosome-related genes

In melanocytes, a melanosome's primary purpose is to package and deliver melanin to keratinocytes [13]. A total of 11/13 genes showed differential expression by ≥1.3 fold in ≥1/3 cell lines (Supplementary Table S4). The binding of miRNAs to mRNA transcripts commonly results in down-regulation of the transcript; however up-regulation is also possible. Interestingly, the overexpression of miR-4731 caused both an up- (MM253 and HT144) and down-regulation (MM96L) of the assessed transcripts to be observed (Supplementary Table S4). For example, in relation to melanoma, the up-regulation of CTSD (cathepsin D) has been suggested to be involved in the malignant transformation and progression of melanocytic tumors [14]. *RAB7A* was also found to be both up- (MM253) and down-regulated (MM96L) upon overexpression of miR-4731 (Supplementary Table S4). RAB7A (a member of the RAS oncogene family) has recently been shown to control melanoma progression and the effect of RAB7A is known to be gene dosage dependent [15]. To further support the effect of miR-4731 expression these melanosome-related genes, we next assessed the expression of other key members of this process (*FSCN1*, *MLPH*, *RAB27A*, and *TYR*). Given these melanosome-related genes were not present in the pull-down data, any observed expression differences as a result of miR-4731 overexpression must be considered as an indirect effect. Nevertheless, *FSCN1*, *MLPH*, *RAB27A*, and *TYR* all showed fold changes ≥1.3 fold in 1/3 (*FSCN1*) and 2/3 (*MLPH*, *RAB27A*, and *TYR*) cell lines (Supplementary Table S6). These additional data strengthen the association of the other confirmed melanosome-related genes (Supplementary Table S4). The highest observed fold changes were for *MLPH* (or melanophilin) with 8.2 and 1.7 down-regulation in HT144 and MM96L respectively. MLPH is known to bind directly to both RAB27A and MYO5A (miR-4731 target gene; Supplementary Table S4), all of which are considered integral components of melanosome transport [16].

#### Cell cycle pathway genes

**Figure 3:**
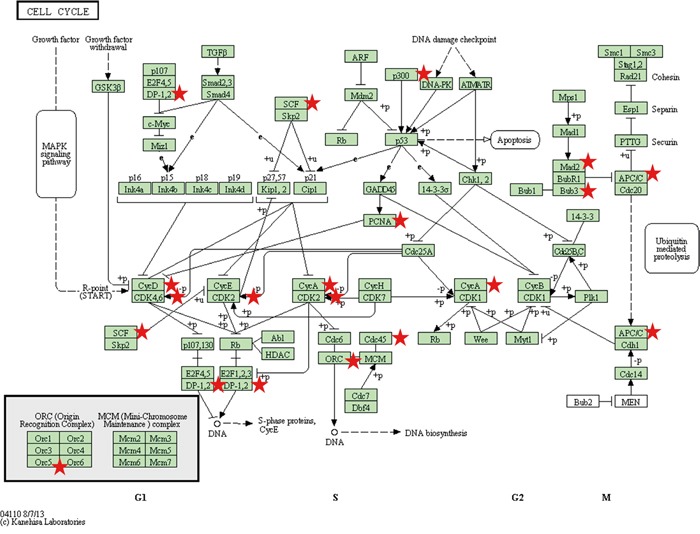
The ‘Cell Cycle’ KEGG pathway is represented with miR-4731 target genes (red stars) This highlights that miR-4731 has the potential to play a critical role in modulating cell cycle progression.

According to Hanahan and Weinberg, the most fundamental trait of cancer cells is their inherent ability to sustain chronic proliferation which, in part, can be attributed to defects in the cell cycle [17]. In our dataset, we confirmed the strongest associated genes, present in 3/3 cell lines, were *CCNA2*, *ORC5L*, and *PCNA* (Supplementary Table S4). *CCNA2* and *PCNA* have been identified as potential biomarkers for melanoma based upon a comprehensive assessment of publically available gene expression datasets [18]. Moreover, in a systematic review of melanoma immunohistochemical (IHC) and gene microarray studies, increased PCNA expression was consistently identified as being associated with worse clinical outcome in melanoma patients [19]. In 2/3 cell lines, *BUB3, CDC45L, CDK4,* and *SKP1A* were also found to be down-regulated. In total, 13/13 genes were down-regulated in ≥1.3 fold in ≥1/3 cell lines (Supplementary Table S4). In the KEGG ‘cell cycle’ pathway shown in Figure 3, the miR-4731 target genes (red star) are represented at almost every critical stage of the cell cycle – indicating that the dysregulation of miR-4731 could be critical to its regulation. A greater in depth analysis is therefore warranted to elucidate this hypothesis.

### Oxidative phosphorylation genes

Oxidative phosphorylation is an important biological process. Recently, dysfunctional oxidative phosphorylation has been shown to be driven by the commonly mutated BRAF oncogene (V600E), allowing malignant melanoma cells to become addicted to glycolysis [20]. Subsequent qRT-PCR showed that 11/15 genes were down-regulated ≥1.3 fold in ≥1/3 cell lines (Supplementary Table S4). However, given that the highest fold-change was −1.6, it is likely that miR-4731 regulation of the pathway is of a relatively minor nature.

### The synovial sarcoma X breakpoint family is regulated by miR-4731

The synovial sarcoma X breakpoint family (SSX) is a cluster of cancer/testis (CT) antigens that have restricted expression in germ cells and is reactivated in tumours [21]. Given this selective expression, particularly in melanoma, it has long been suggested that they would be prime candidates for immunotherapeutic treatment which has now recently been shown *in vitro* to have promising results [22]. SSX2 was the first to be identified as being expressed in melanoma tumours (50%) [23] and in a follow-up study Gure *et al* [24] discovered that the other SSX family members were also expressed at varying levels in both tumour and melanoma patient-derived serum. SSX expression has also been found to be an early event in melanocytic neoplasia with 2/24 naevi showing nuclear staining [25].

**Figure 4:**
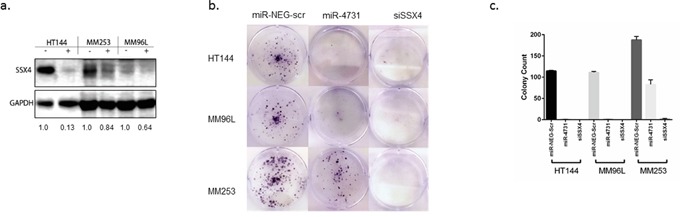
**a.** SSX4 expression is reduced following transient transfection of miR-4731-5p (miR-4731) mimic (5 nM) as compared to miR-Neg-scr control. +/− indicates overexpression of the miR-4731 mimic (+) or miR-Neg-scr control (-). Numbers indicate relative fold change. SSX4 (~22 kDa) and GAPDH (~37 kDa) were run and quantified on the same gel. **b.** HT144, MM96L, and MM253 and were transiently transfected with 5 nM of siSSX4, miR-4731 mimic, and a negative control (miR-Neg-scr) with colonies stained with crystal violet 10-14 days post transfection. This assay was repeated twice in triplicate and representative results are shown. **c.** Graphical representation of colony counts in replicate wells performed for each respective colony assay.

Indications that the SSX family members (SSX1-4, SSX4B, and SSX5) were regulated by miR-4731 can be observed in Supplementary Table S2, which shows that these genes were pulled down in 3/3 cell lines with fold changes ranging from 2.3 to 56.86. As a general rule, the fold change enrichment does not necessarily reflect the biological consequence of the binding of the biotinylated miRNA, however in these cases, all transcripts (except SSX5) were associated as being under transcriptional control of miR-4731. Importantly, knockdown of SSX4 and SSX4B by siRNAs has previously been shown to result in marked reduction (~50%) in 2D colony formation in SK-MEL-37 melanoma cells [26]. Strikingly, cell proliferation and migration were unaffected by the lack of SSX4 or SSX4B expression [26]. Furthermore, in A375 cells, induction of SSX2 expression using a lentiviral construct (above endogenous tumour levels) led to a reduction of cell viability and colony formation, and induced cell cycle G1 checkpoint arrest [27]. SSX2 expression was further found to induce DNA damage response which promoted genomic instability [27]. The authors also showed that knockdown of SSX2 expression had a similar effect to the overexpressed constructs, with cell growth being diminished [27]. Therefore, based on these data, the authors concluded that melanoma appears dependent on an optimal level of SSX2 expression [27]. With these data in mind, we selected SSX4 as a representative of the SSX family for further investigation. In the same 3 melanoma cell lines, overexpression of miR-4731 mimic resulted in a 1.2-7.5 fold reduction of SSX4 protein expression relative to miR-NEG-scr (Figure 4a). In keeping with previously published observations, knockdown of SSX4 via a siRNA pool showed a complete ablation of 2D colony formation (Figure 4b–4c) in all 3 cell lines. Importantly, overexpression of miR-4731 mimic had the same effect on colony formation as SSX4 knockdown in HT144 and MM96L, with MM253 (Figure 4b–4c) showing a 1.7 fold decrease (~41% reduction), similar to the prior observation in SK-MEL-37 [26]. The lesser effect observed in MM253 matches the SSX4 protein expression (1.2 fold decrease) (Figure 4a). Interestingly, despite the dramatic reduction in 2D colony formation, there was no significant change in cell viability (data not shown). These data mirror the previous study by Caballero *et al.* [26] and strongly suggest that miR-4731 modulates this via its regulation of SSX4. Although the caveat exists that the level of miR-4731 overexpression may be higher than that seen *in vivo*, we would argue that given the observed effect on the majority of the transcripts assessed, especially SSX4 at the protein level, that this level of overexpression, may in the future actually have therapeutic relevance – i.e. enhancing the tumour suppressive role of miR-4731. We therefore speculate that since the expression of miR-4731 decreases upon distal metastatic spread [2], it could be inferred that SSX4 protein levels would also increase, leading to enhanced tumour formation.

### Conclusions

What is apparent from the GSEA of the pulled-down miR-4731 target genes is that a single miRNA has the potential to have multiple effects, being involved in regulating genes involved in key pathways/processes related to melanocyte biology and/or melanoma. The identification of these enriched processes was enhanced by the use of high-throughput techniques such as the synthetic biotinylated miRNA-duplex pull-down procedure used here [6, 7] combined with an integrated network analysis programs such as STRING [12] which provided novel insights into our dataset. The validated pathways that we have highlighted, together with the commonly pulled down genes such as the SSX family, provide a significant insight into the role of miR-4731 in melanomagenesis. In our previous publication [2], we found that the expression of miR-4731 was significantly lower in tissues derived from stage IV patients compared with stage III tissues. These data suggests that miR-4731 may have a tumour suppressive function (with loss of a tumour suppressor being favourable to the metastatic tumour). Together with the functionally validated effect of miR-4731 on SSX4 protein expression, we have many other gene targets to bolster this notion. For example, the target gene *CDK4*, is well known as a potent oncogene in melanoma which is normally regulated via the tumour suppressor CDKN2A (p16). Upon overexpression of miR-4731, we have shown that CDK4 expression levels are reduced in 2/3 cell lines which provide further support of miR-4731 having a tumour suppressive role. In the future, we may see melanoma-related miRNAs like miR-4731 being used for therapeutic purposes – i.e. specifically targeted to melanoma with the aim of restoring miRNA levels, and in turn their interacting partners, to homeostasis. Finally, identification of target genes of ‘tissue-specific’ miRNAs like miR-4731 may allow a greater understanding of the tumourigenic process, unique to each cancer type. Armed with this knowledge, we envisage that better tailored therapeutic options could be devised.

## MATERIALS AND METHODS

### Cell culture and total RNA extraction

All melanoma cell lines (Supplementary Table S1) have been previously described [1, 28, 29].

Cell lines were cultured in RPMI-1640 (#31800-089, Life Technologies, Foster City, CA, USA) supplemented with 10% fetal bovine serum (FBS) (Life Technologies), HEPES, 100 U/ ml penicillin and 100 μg/ml streptomycin (Life Technologies), and incubated at 37°C (5% CO_2_). All cell lines were periodically authenticated via short tandem repeat profiling according to the manufacturer's instructions (AmpFISTR Profiler Plus ID kit; Life Technologies).

Cells were harvested from the plate and column-purified using the miRNeasy Kit (QIAGEN, Hilden, Germany) according to the manufacturer's instructions. Ethical approval for the study was granted by the QIMR Berghofer's Human Research Ethics Committee (HREC), approval number P1237.

### miScript quantitative RT-PCR validation

Cell lines were reverse transcribed using miScript II RT Kits (QIAGEN) according to the manufacturer's instructions. Real-time PCR was subsequently performed with a miScript SYBR Green PCR Kit (QIAGEN) using the 7900HT Fast Real Time PCR System (Life Technologies). Data were analysed in Microsoft Excel using the ΔCT method compared to RNU6 which was assessed in each sample. ΔCT values were plotted and statistical analysis performed in GraphPad Prism (v6.04).

### Biotin pull-downs and microarray hybridizations and data analysis

Melanoma cell lines (MM96L, MM253 and HT144; all were BRAF V600E mutant positive) were selected based on their high transfection ability together with having endogenous miR-4731 expression. Synthetic biotinylated microRNA-duplexes were designed for miR-4731 (Sequence 1: /5Phos/rUrGrCrUrGrGrGrGrGrCrCrArCrArUrGrArGrUrGrUrG/3Bio/ Sequence 2: /5Phos/rCrArCrArCrArArGrUrGrGrCrCrCrCrCrArArCrArUrU) along with a scrambled control (Sequence 1: /5Phos/rUrArUrCrCrCrCrUrUrUrGrCrCrUrGrCrUrUrUrUrCrC/3Bio/ Sequence 2: /5Phos/rUrArArGrCrUrArGrArCrCrGrGrArGrGrArGrGrGrC) according to specifications detailed in the methodology devised by Cloonan and collegues [6, 7] (and optimised for melanoma cell lines [1]) and purchased from Integrated DNA Technologies (Coralville, USA). Microarray hybridizations were carried out as previously described (1).

In an effort to be inclusive, gene lists from each cell line were generated if the transcript occurred at an increased level (≥1.15 fold; ranged up to 51 fold) in the miR-4731 ‘pull-down’ as compared to the Neg-Scr. Next, each gene list was compared using a Venn diagram in GeneSpring GX 12.5 (Agilent Technologies) to identify overlapping transcripts. If a transcript was present in 3/3 cell lines, it was carried forward into subsequent analysis (Supplementary Figure S1).

### Network analysis

Transcripts present in 3/3 cell lines were used to identify relationships amongst each gene along with enrichment for key signalling pathways. First, obsolete accession numbers, identified using *Batch Entrez* (http://www.ncbi.nlm.nih.gov/sites/batchentrez) were removed from the dataset as they were no longer associated with a *bona fide* gene. A list of unique gene names was then generated which could be imported into STRING v9.1 (http://string-db.org/) which allowed for the identification and visualisation of known and predicted protein-protein interactions [12]. Enrichment for significant pathways (KEGG; http://www.kegg.jp/kegg/pathway.html) and GO terms (Gene Ontology; http://geneontology.org/) were also performed using STRING v9.1. False discovery rates (FDR) (i.e. corrected Bonferroni *P*-value) were applied against a genome-wide level, to the associated pathway.

### microRNA target site prediction

All transcripts that were present in 3/3 cell lines were confirmed to be a potential target gene using the binding algorithm miRanda v3.3a [30] downloaded from http://www.microrna.org/microrna/getDownloads.do (miRan da-Aug2010 version). FASTA formatted sequences were obtained for miR-4731 from miRBASE (http://www.mirbase.org/) and target genes were identified using a batch query from the NCBI (http://www.ncbi.nlm.nih.gov/sites/batchentrez). The program was run using default conditions with exceptions (binding threshold was set to 100). TargetScan [31] (Release 6.2: June 2012), miRDB [32], TargetMiner [33], and DIANA-microT-CDS [34] predicted transcripts were identified via online databases and compared to miRanda v3.3a.

### Criteria for selecting genes for qRT-PCR validation

First, the host gene of miR-4731, *PMP22* was selected to confirm if it was under transcriptional control of miR-4731. It is important to note that *PMP22* was also a pull-down target in 3/3 cell lines. Second, all genes that had ≥2 fold enrichment in 3/3 cell lines (n=37) were selected. Third following the input of the 1092 unique Gene Names into STRING v9.1 (n=1076 were identified and present in the database), gene set enrichment was performed (GSEA) for KEGG pathways (cell cycle and oxidative phosphorylation), and the gene ontology (GO) terms: ‘Biological Process’, ‘Molecular Function’, and ‘Cellular Components’ (melanosomes). Significant ‘pathways’ were selected based upon *a priori* relevance to melanoma and/or melanocyte biology. The significant pathways selected (‘cell cycle’, ‘oxidation phosphorylation’, and ‘melanosomes’) had further restrictions imposed in an attempt to be more stringent. i.e. all genes present in these pathways must have been pulled down in ≥1.5 fold, in ≥2/3 cell lines (n=43). In addition, considering the importance of the melanosome in melanomagenesis, we selected additional genes (not present in the pull-down) relevant to this the melanosome (*FSCN1*, *MLPH*, *RAB27A*, and *TYR*), to observe the effect of miR-4731 overexpression.

### Transient transfection of miR-4731 mimic and negative scrambled control in melanoma cell lines

Transfection conditions have been previously described [1]. miR-4731-5p mimic (#MSY0019853), and Negative Allstars control (#1027280; miR-NEG-scr) were purchased from QIAGEN. A final concentration of 5 nM of mimic and negative control were reverse-transfected into MM96L, MM253 and HT144 (30,000 cells/6-well) using Lipofectamine® RNAiMAX (Life Technologies) and harvested for RNA at 72 h. RNA and protein was extracted as previously described [1]. Each biological sample was a pool of three transfected wells from a 6-well plate. Two independent experiments were performed.

### Custom RT^2^ profiler PCR array

First, 400 ng of RNA from duplicate biological samples (individual transfections), underwent cDNA synthesis using the RT^2^ First Strand Kit (QIAGEN) followed by qRT-PCR using the RT^2^ SYBR^®^ Green qPCR Mastermix (QIAGEN) and the Custom RT^2^ Profiler PCR Array (QIAGEN) which contained the pre-designed primer assays for all selected pull-down transcripts (n=83) (Supplementary Table S7). The parameters were as per manufacturer's instructions. The array also had the endogenous controls *HPRT1*, *RPLP0*, *ACTB* (also a target of miR-4731), *GAPDH*, *B2M* and *TFRC* together with controls for assessing gDNA contamination and technical reproducibility (triplicate wells of reverse-transcription controls (RTC) and a positive PCR control (PPC)). These controls allow for testing of the PCR efficiency, with the replicates testing for inter-well, and intra-plate consistency. Each 384 well plate had duplicate samples for the overexpression of miR-4731 and the negative control (i.e. 4 biological samples per plate). Real-Time PCR was performed using the ViiA™ 7 Real-Time PCR System (Life Technologies) as per manufacturer's instructions. CT values were extracted using ViiA™ 7 Software v1.2.4 (Life Technologies) and analysed using the RT^2^ Profiler PCR Array Data Analysis software (QIAGEN).

### Western blot and protein analysis

Samples (40 μg total protein) were resolved on 4–15% Mini-PROTEAN TGX gels (Bio-Rad, Hercules, USA) and transferred to PVDF membranes using a Trans-Blot®Turbo™ (Bio-Rad, Hercules, USA). The following antibodies were used to detect SSX4 (#ab172215; Abcam) and GAPDH (#2275-PC-100, R&D systems) at 1:1000 and 1:5000 dilutions respectively along with a HRP-linked anti-mouse secondary at 1:10,000 (Thermo Fisher) or anti-rabbit secondary at 1:7500 (Merck Millipore). Enhanced chemiluminescence (ECL) detection of antibody binding was quantified using the C-Digit scanner and Image Studio software (Licor).

### 2D Colony assays

Melanoma cell lines (MM96L, MM253, and HT144) were reverse transfected with a SSX4 siRNA (siSSX4; # 1027416, siRNA #4, FlexiTube GeneSolution GS6759), a miR-4731-5p mimic (#MSY0019853), and a Negative Allstars control (#1027280; miR-NEG-scr) purchased from QIAGEN as described and seeded at a density of 1000 cells/well into a 6-well plate. Colonies were stained with crystal violet 10-14 days post transfection using standard methodology. Triplicate wells for each transfection were performed in two independent experiments. Colonies were counted using Clono-Counter software [35].

### Cell viability assays

Cell viability assays were performed and determined using a modified sulforhodamine B (SRB; Sigma, St Louis, USA) assay [36]. Briefly, miRNA mimic and miR-NEG-scr were reverse-transfected with melanoma cell lines and seeded into a 96-well plate (6 wells/transfection) then incubated at 37°C with 5% CO2 for 96 h. Plates were fixed on day 4 with methylated spirits prior to performing the SRB assay and read at 564 nm using a plate reader (Molecular Devices, Sunnyvale, USA). Each experiment (6 wells) was performed in duplicate using independent transfections.

## SUPPLEMENTARY MATERIALS FIGURES AND TABLES
















