# Supplementary material for: The ‘melanoma-enriched’ microRNA miR-4731-5p acts as a tumour suppressor
Source: Oncotarget. 2016 Jun 16;7(31):49677–87. doi: 10.18632/oncotarget.10109 (PMC5226538; doi:10.18632/oncotarget.10109)
Supplement: Supplementary file 1 [file oncotarget-07-49677-s001.pdf]

The “melanoma-enriched” microRNA miR-4731-5p acts as a tumour suppressor

REFERENCES

1. Stark MS, Bonazzi VF, Boyle GM, Palmer JM, Symmons J, Lanagan CM, Schmidt CW, Herington AC, Ballotti R, Pollock PM, Hayward NK. miR-514a regulates the tumour suppressor NF1 and modulates BRAFi sensitivity in melanoma. Oncotarget. 2015; 6:17753-17763.
2. Franceschini A, Szklarczyk D, Frankild S, Kuhn M, Simonovic M, Roth A, Lin J, Minguez P, Bork P, von Mering C, Jensen LJ. STRING v9.1: protein-protein interaction networks, with increased coverage and integration. Nucleic Acids Res. 2013; 41:D808-815.

SUPPLEMENTARY FIGURES AND TABLES

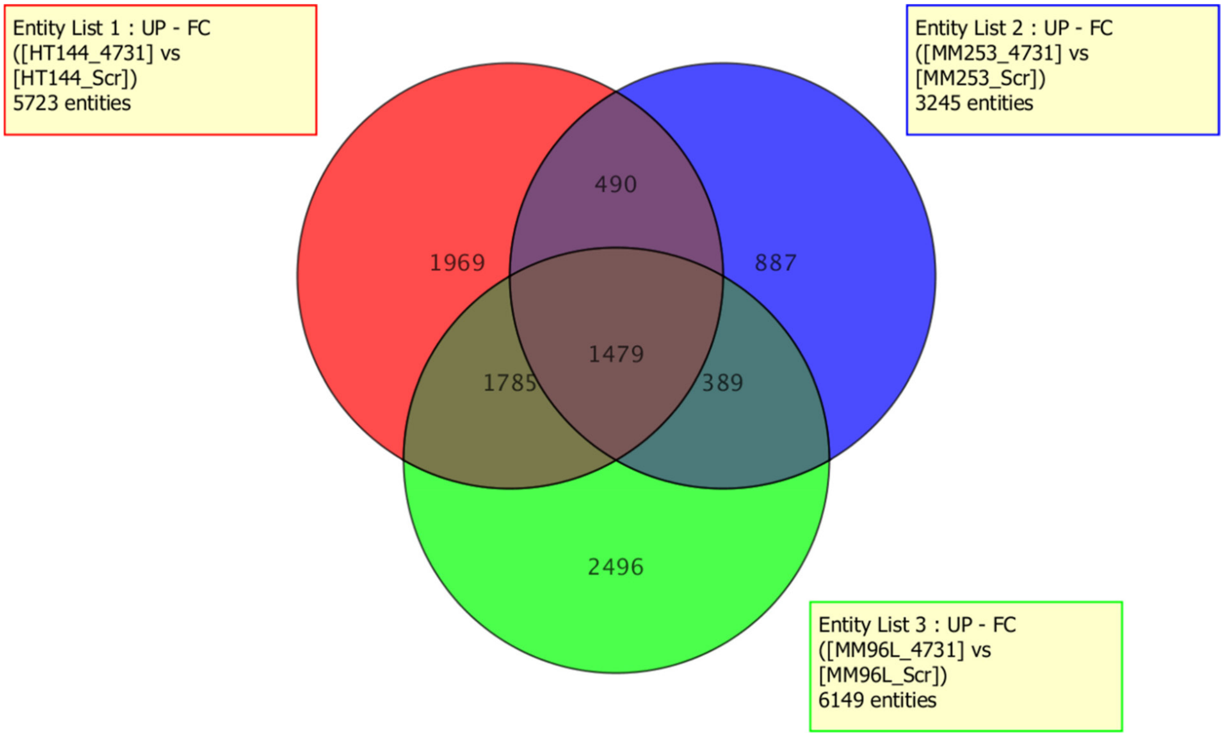

Supplementary Figure S1: Venn diagram showing the overlap of genes pulled-down in 3/3 cell lines.

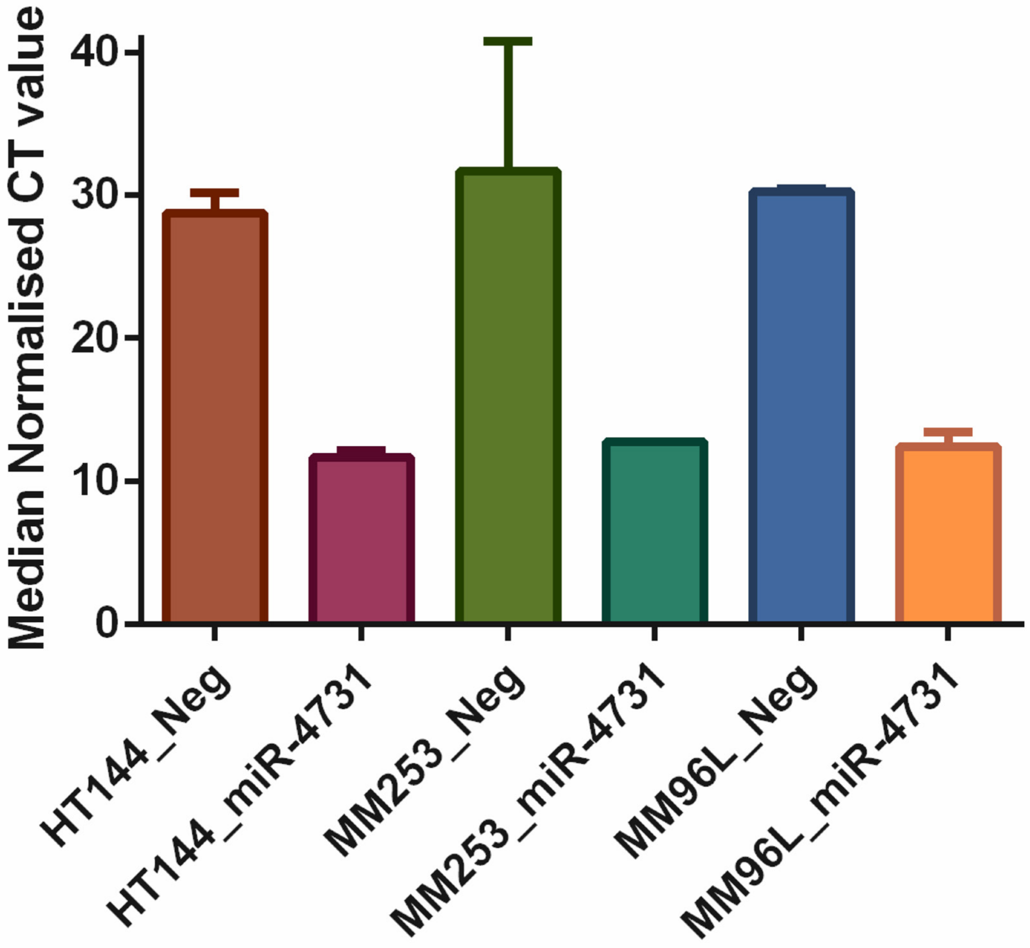

Supplementary Figure S2: Endogenous expression (Neg) of miR-4731 is low in MM96L, MM253, and HT144 and is highly expressed upon transient overexpression with a miR-4731 mimic (miR-4731).

Supplementary Table S1: Summary of all cell line names and associated tissue type that were used in the discovery (microarray) [1] and validation cohorts (qRT-PCR)

See Supplementary File 1

Supplementary Table S2: Unique transcripts common in 3/3 cell lines derived from GeneSpring 12.5 GX analysis. 'PULL-DOWN DATA' shows the respective fold changes in each cell line relative to the negative control mimic

See Supplementary File 2

Supplementary Table S3: Significant (corrected *P*≤0.05) KEGG pathways and GO Terms (Biological Process, Molecular Function, and Cellular Component) were found following GSEA using STRING v9.1 analysis [2]

See Supplementary File 3

Supplementary Table S4: The 'RATIONALE FOR VALIDATION' is noted together with the 'qRT-PCR VALIDATION' fold changes for each cell line

See Supplementary File 4

Supplementary Table S5: Summarises the genes that were; down regulated in 3/3 (n=19), 2/3 (n=12) and 1/3 (n=19); both up and down regulated (n=5); up regulated (n=5); and no observable change at the mRNA level (≤1.2 fold up or down regulated) (n=21). A relationship (based upon Pubmatrix searches) with 'Melanoma' and 'Cancer' is noted as 'Novel' or Y (Yes or Known)

See Supplementary File 5

Supplementary Table S6: Respective fold-changes observed in MM96L, MM253, and HT144 in melanosome-related genes upon overexpression with miR-4731 (relative to negative control). All genes list are not direct targets of miR-4731, as such are downstream of its regulation

See Supplementary File 6

Supplementary Table S7: Assay IDs (QIAGEN) used for qRT-PCR validation

See Supplementary File 7
